# Risk of anxiety disorders in men with prostate cancer: a national cohort study

**DOI:** 10.1093/jncics/pkae087

**Published:** 2024-09-14

**Authors:** Casey Crump, Pär Stattin, James D Brooks, Jan Sundquist, Kristina Sundquist, Weiva Sieh

**Affiliations:** Departments of Family and Community Medicine and of Epidemiology, The University of Texas Health Science Center, Houston, TX, USA; Department of Surgical Sciences, Uppsala University, Uppsala, Sweden; Department of Urology, Stanford University School of Medicine, Stanford, CA, USA; Center for Primary Health Care Research, Department of Clinical Sciences, Lund University, Malmö, Sweden; Center for Primary Health Care Research, Department of Clinical Sciences, Lund University, Malmö, Sweden; Department of Epidemiology, The University of Texas MD Anderson Cancer Center, Houston, TX, USA

## Abstract

**Background:**

Men with prostate cancer (PC) may experience significant psychosocial distress from physical symptoms, treatment side effects, or fear of recurrence. However, little is known about the long-term risk of anxiety disorders in men with PC.

**Methods:**

A national cohort study was conducted of 180 189 men diagnosed with PC during 1998-2017 and 1 801 890 age-matched population-based control men in Sweden. Anxiety disorders were ascertained from nationwide outpatient and inpatient records through 2018. Cox regression was used to estimate hazard ratios (HRs) while adjusting for sociodemographic factors and prior psychiatric disorders. Subanalyses explored differences by PC treatment during 2005-2017.

**Results:**

In 7.8 million person-years of follow-up, 94 387 (5%) men were diagnosed with anxiety disorders. Men with high-risk PC had a nearly 2-fold higher risk of anxiety disorders than control men without PC (adjusted HR = 1.96, 95% CI = 1.87 to 2.05). This risk was highest in the first 3 months after PC diagnosis (adjusted HR = 2.99, 95% CI = 2.49 to 3.59) but remained significantly elevated 10 or more years later (adjusted HR = 1.53, 95% CI = 1.35 to 1.74). Those treated only with androgen deprivation therapy (ADT) had the highest risk of anxiety disorders (adjusted HR = 2.08, 95% CI = 1.93 to 2.25). Men with low- or intermediate-risk PC had a modestly increased risk (adjusted HR = 1.39, 95% CI = 1.34 to 1.44).

**Conclusions:**

In this large national cohort, men with PC had substantially increased risk of anxiety disorders, especially those with high-risk PC and treated only with ADT. Men with PC need close monitoring for timely detection and treatment of anxiety symptoms, particularly shortly after PC diagnosis.

## Graphical Abstract

**Graphical Abstract:**
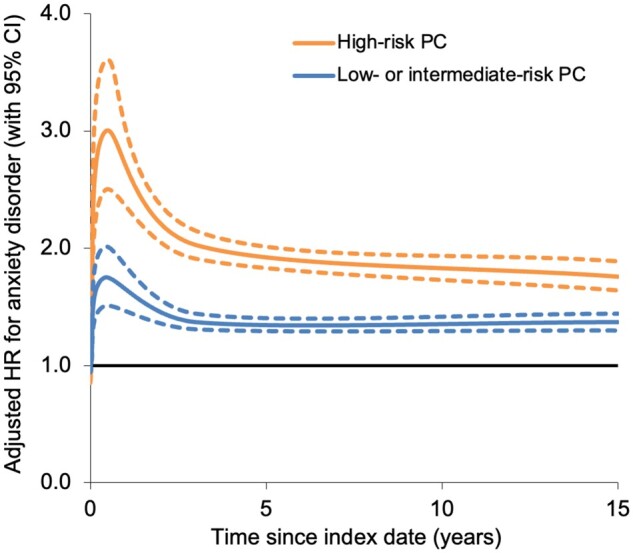

Prostate cancer (PC) is the most commonly diagnosed cancer among men in most countries, with more than 1.5 million new diagnoses worldwide each year (1,2). Men with PC may experience significant psychosocial distress because of physical symptoms, treatment side effects, or fear of recurrence (3) and have an increased risk for depression (4-6). Anxiety disorders often co-occur or have overlapping symptoms with depression (7), and yet also have unique symptoms characterized by uncontrollable feelings of anxiety, fear, or avoidance behaviors that may impair overall function and quality of life (8-10). Anxiety disorders are the most common mental disorders in the United States (11) and globally (12), but they are understudied in men with PC. A better understanding of the long-term risk of anxiety disorders is needed to guide psychosocial interventions and improve outcomes in the growing number of PC survivors.

A recent meta-analysis of 56 small clinical studies (mean sample size <500) reported that 17% of men with PC have “significant anxiety symptoms” (13). However, those studies lacked a comparison group of men without PC and clinical diagnoses of anxiety disorders; thus, the impact of PC on risk of anxiety disorders is still largely unknown. To our knowledge, no population-based cohort studies have assessed the long-term risk of anxiety disorders in men with vs without PC, while controlling for potential confounders. We sought to address these knowledge gaps using nationwide data in Sweden. Our goals were to determine the long-term risks of anxiety disorders among men with PC of different prognoses in a large population-based cohort, identify periods of heightened risk after PC diagnosis, and assess for potential age-specific differences. We hypothesized that men of all ages with aggressive PC have increased long-term risk of anxiety disorders.

## Methods

### Study population and PC ascertainment

This study was based on a cohort previously included in studies of other outcomes (4,14,15). In the National Prostate Cancer Register (NPCR) of Sweden, we identified 183 495 men who were diagnosed with PC during 1998-2017 (4,14,15). NPCR is a clinical cancer register with the aim to collect, analyze, and report data as a basis for quality assurance for PC care, including adherence to national guidelines (16-18). The NPCR includes 98% of all incident PC cases since 1998 compared with the Swedish National Cancer Register, which has nationwide mandated reporting (19). It contains information on cancer characteristics including tumor grade according to Gleason score; disease stage based on the tumor, nodes, metastasis (TNM) classification; and PSA level at diagnosis (20-22). We excluded 3306 (2%) men who had missing data for any of these characteristics, leaving 180 189 (98%) men for analysis as in prior studies (4,14,15).

PC risk groups were defined at the time of diagnosis based on a modification of the National Comprehensive Cancer Network (NCCN) Practice Guidelines criteria as used by the NPCR (19, 23). Low-risk PC was defined by clinical local stage T1-T2, Gleason score 2-6, and PSA less than 10 ng/mL, and intermediate-risk PC by T1-T2 with Gleason score 7 and/or PSA 10 to less than 20 ng/mL. High-risk PC was defined by clinical stage T3 or T4, Gleason score of 8 or higher, and/or PSA of at least 20 ng/mL at the time of diagnosis and was further stratified as locally advanced (stage T3 and PSA 20 to <50 ng/mL), very advanced/regionally metastatic (stage T4 and/or N1 and/or PSA 50 to <100 ng/mL in the absence of distant metastases [M0 or Mx]), or distant metastases (stage M1 and/or PSA ≥100 ng/mL) (19,23). Primary treatment within 6 months after diagnosis also was identified from the NPCR. Androgen deprivation therapy (ADT) was further identified using Anatomical Therapeutic Chemical codes L02AE (gonadotropin-releasing hormone [GnRH] analogues), L02BB (anti-androgens), and L02BX (other hormone antagonists) in the Swedish Prescribed Drug Register, which contains all medication prescriptions dispensed nationwide since July 1, 2005.

The Swedish Total Population Register includes data on the composition of the entire Swedish population since 1968 (24). Using this register, each PC case was matched to 10 men randomly sampled from the general population who had the same birth year and month and were living in Sweden on the date of PC diagnosis for the respective case (ie, index date) (4,14). This study was approved by the Regional Ethical Review Board in Lund, Sweden (No. 2012/795 and later amendments). Participant consent was not required because this study used only pseudonymized registry-based secondary data.

### Anxiety disorders ascertainment

The primary outcome was the earliest diagnosis of anxiety disorders, which were ascertained from the index date (respective cases’ PC diagnosis date) through December 31, 2018. Anxiety disorders were identified using the *International Classification of Diseases, 10th Revision* (ICD-10) codes F40-F42 in the Swedish In-Patient and Out-Patient Registers and primary care records. The In-Patient Register includes all primary and secondary hospital discharge diagnoses with 86% coverage of the Swedish population starting in 1973 and 100% coverage from 1987 onward (14,25). The Swedish Out-Patient Register contains all diagnoses from specialty clinics with approximately 87% nationwide coverage starting in 2001 (26). Primary care diagnoses (27) were available for 20% of the Swedish population starting in 1998, 45% starting in 2001, and 90% starting in 2008 and onward (4,14,15,28).

### Covariates

Other characteristics that may be associated with PC and anxiety disorders were identified using Swedish national census and health registry data. Covariates included birth date (continuous and categorical by decade), birth country (Sweden/other), marital status (married/not married), education level (compulsory high school or less [≤9 years], practical or theoretical high school [10-12 years], college or higher [>12 years]), income (quartiles), region (large cities, other/Southern, other/Northern, unknown), and prior history of major depression, anxiety disorders, bipolar disorder, or schizophrenia (each ascertained from 1973 up to the index date and modeled as a separate covariate). Psychiatric disorders were ascertained from the Swedish In-Patient and Out-Patient Registers and primary care records using ICD-10 codes (major depression, F32-F33; anxiety disorders, F40-F42; bipolar disorder, F31; schizophrenia, F20). All covariates were more than 96% complete. Missing data were modeled as a separate category and had minimal influence on risk estimates because of their rarity (14).

### Statistical analysis

Cox regression was used to compute hazard ratios (HRs) and 95% confidence intervals (CIs) for anxiety disorders in men with PC compared with matched controls, while adjusting for covariates and stratifying on matched sets. The observation period for each study participant (PC cases and their matched controls) began at the index date (ie, date of PC diagnosis for the respective case) and ended at the date of earliest diagnosis of anxiety disorders or December 31, 2018, whichever came first. Men were censored at the date of death (n = 556 917) or of emigration (n = 15 099). In a secondary analysis, we also explored the association between PC and “new-onset anxiety disorders” after excluding 7184 (4%) PC cases and 81 331 (5%) controls who had a prior registration of any anxiety disorder before the index date. The proportional hazards assumption was evaluated by examining log-log survival plots and was satisfied in each model.

To assess periods of susceptibility, anxiety disorders were assessed within specific time intervals after PC diagnosis (<3, 3 to <12 months; 1 to <2, 2 to <5, 5 to <10, ≥10 years) in separate models. Anxiety disorders also were further stratified by primary treatment modality (ADT only; radical radiotherapy with or without adjuvant ADT; radical prostatectomy; or radical prostatectomy followed by radiotherapy) using treatment data during 2005-2017, compared with controls. ADT was further examined as GnRH analogues vs anti-androgen monotherapy.

In exploratory analyses, age-specific differences were assessed by stratifying on age at the index date (<55, 55-64, 65-74, 75-84, ≥85 years) while adjusting for age as a continuous variable within each stratum. To assess for temporal changes in diagnostic or coding practices, we explored associations between high-risk PC and anxiety disorders after stratifying on calendar year of PC diagnosis (1998-2004, 2005-2009, 2010-2017). A secondary analysis also was performed after restricting the outcome to anxiety disorders diagnosed in specialty care. All statistical tests were 2-sided and used a significance level of .05. All analyses were conducted using Stata version 16.1.

## Results

Of the 180 189 men with PC, 56% had low- or intermediate-risk PC and 44% had high-risk PC. Men with low- or intermediate-risk PC had a median age of 67 years (interquartile range [IQR] = 62-73) at diagnosis and a median follow-up time of 7 years (IQR = 4-11). Men with high-risk PC had a median age of 75 years (IQR = 68-81) at diagnosis and a median follow-up time of 4 years (IQR = 2-8). Control men without PC had a median follow-up time of 5 years (IQR = 2-9).

In 7.8 million person-years of follow-up, a total of 94 387 (5%) men (with or without PC) were diagnosed with anxiety disorders. Of these diagnoses, 75% were anxiety disorders not otherwise specified, whereas 12% were specified as generalized anxiety disorder, 8% as panic disorder, 3% as specific phobic disorders, and 2% were other or mixed anxiety disorders. At 5 years of follow-up, the cumulative incidence of anxiety disorders was 4% among men with PC and 3% among control men. The median age at registration of anxiety disorders was 72 years in men with low- or intermediate-risk PC, 78 years in men with high-risk PC, and 75 years in controls.

Table 1 shows characteristics of men with PC, controls, and all men with anxiety disorders. Men with PC were more likely than controls to be married. Men with high-risk PC had lower education and income levels than controls, whereas men with low- or intermediate-risk PC had higher education or income levels. Men with anxiety disorders were younger and more likely to be unmarried, live in large cities, or have a prior diagnosis of psychiatric disorders.

**Table 1.** Characteristics of men with prostate cancer and control men, 1998-2018, Sweden

	**High-risk PC** ^a^	**Low- or intermediate-risk PC** ^b^	Controls	Anxiety disorders
	**N = 78** **951**	**N = 101** **238**	**N = 1** **801** **890**	**N = 94** **387**
	n (%)	n (%)	n (%)	n (%)
**Age at index date (years)**				
<55	1372 (2)	5728 (6)	71 000 (4)	5611 (6)
55-64	10 791 (14)	32 406 (32)	431 970 (24)	26 242 (28)
65-74	27 258 (34)	45 054 (44)	723 120 (40)	37 798 (40)
75-84	30 272 (38)	16 356 (16)	466 280 (26)	20 784 (22)
≥85	9258 (12)	1694 (2)	109 520 (6)	3952 (4)
**Sweden-born**	72 846 (92)	92 334 (91)	1 573 042 (87)	82 786 (88)
**Marital status**				
Married	54 933 (70)	72 080 (71)	1 141 069 (63)	57 684 (61)
Not married	24 018 (30)	29 157 (29)	606 285 (34)	36 702 (39)
Unknown	0 (0)	1 (<0.1)	54 536 (3)	1 (<0.1)
**Education (years)**				
≤9	37 659 (48)	32 615 (32)	748 594 (41)	37 675 (40)
10-12	27 165 (34)	40 885 (40)	648 123 (36)	37 513 (40)
>12	14 117 (18)	27 727 (27)	355 450 (20)	19 179 (20)
Unknown	10 (<0.1)	11 (<0.1)	49 723 (3)	1 (<0.1)
**Income (quartile)**				
1st (highest)	15 832 (20)	39 920 (39)	468 355 (26)	22 486 (24)
2nd	21 091 (27)	28 529 (28)	462 783 (26)	28 246 (30)
3rd	22 291 (28)	20 252 (20)	438 894 (24)	27 401 (29)
4th (lowest)	19 694 (25)	12 481 (12)	359 227 (20)	15 976 (17)
Unknown	43 (0.1)	56 (0.1)	72 631 (4)	278 (0.3)
**Region**				
Large cities	34 100 (43)	53 073 (52)	825 039 (46)	54 959 (58)
Other/Southern	29 884 (38)	33 257 (33)	618 625 (34)	25 921 (27)
Other/Northern	14 957 (19)	14 899 (15)	310 444 (17)	13 493 (14)
Unknown	10 (<0.1)	9 (<0.1)	47 782 (3)	14 (<0.1)
**Prior psychiatric disorders**				
Major depression	3167 (4)	5108 (5)	91 107 (5)	21 881 (23)
Anxiety disorder	2814 (4)	4370 (4)	81 331 (5)	31 215 (33)
Bipolar disorder	346 (0.4)	626 (0.6)	11 708 (0.6)	2403 (3)
Schizophrenia	247 (0.3)	235 (0.2)	11 808 (0.7)	1150 (1)

a High-risk PC was defined by clinical stage T3-T4, Gleason score ≥8, and/or PSA ≥20 ng/mL at time of diagnosis. PC = prostate cancer.

b Low-risk PC was defined by clinical stage T1-T2, Gleason score 2-6, and PSA <10 ng/mL, and intermediate-risk PC by clinical stage T1-T2 with Gleason score 7 and/or PSA 10 to <20 ng/mL.

### PC and risk of anxiety disorders

**Figure 1.**
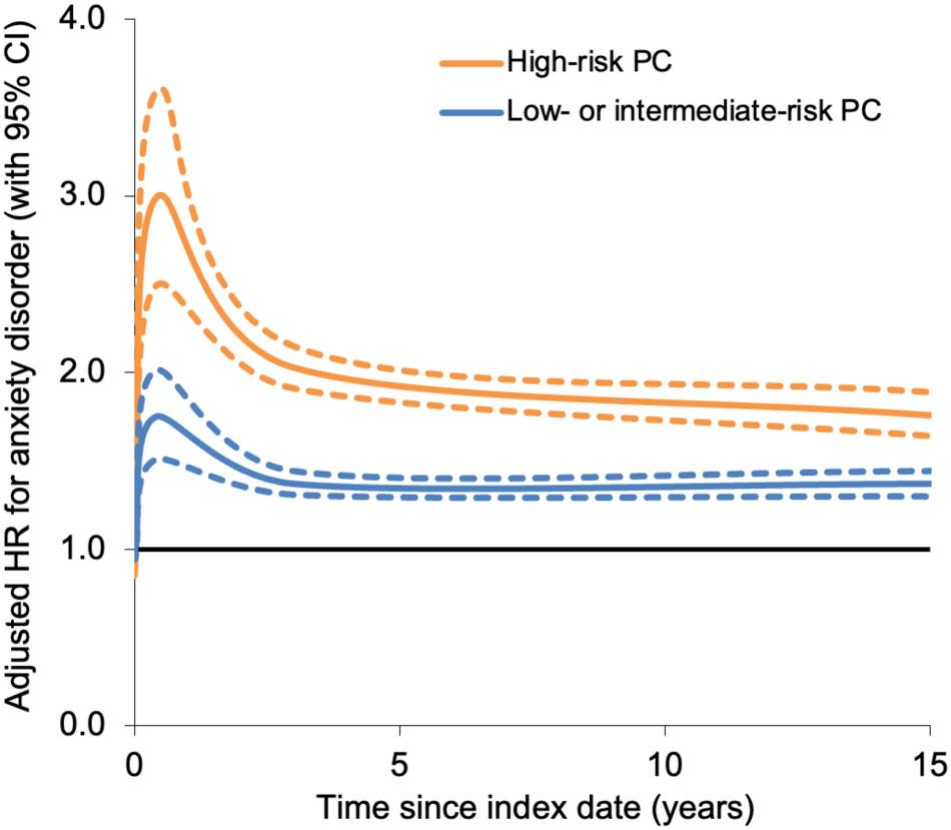
Adjusted HRs for anxiety disorders associated with high-risk PC or low- to intermediate-risk PC by time since index date, 1998-2018, Sweden (dotted lines represent 95% CI).

Across the entire follow-up period, men with high-risk PC had a nearly 2-fold higher risk of anxiety disorders compared with control men without PC (adjusted HR = 1.96, 95% CI = 1.87 to 2.05) (Table 2). This risk was higher in men with very advanced/regionally metastatic disease (adjusted HR = 2.45, 95% CI = 2.20 to 2.72) or distant metastases (adjusted HR = 2.31, 95% CI = 2.03 to 2.62) than those with locally advanced disease (adjusted HR = 1.81, 95% CI = 1.72 to 1.92) (Supplementary Table 1, available online). Risk of anxiety disorders was highest within the first 3 months (adjusted HR = 2.99, 95% CI = 2.49 to 3.59) but remained significantly elevated ≥10 years after PC diagnosis (adjusted HR = 1.53, 95% CI = 1.35 to 1.74) (Table 2). Low- or intermediate-risk PC was associated with a modestly increased risk of anxiety disorders across the entire follow-up period (adjusted HR = 1.39, 95% CI = 1.34 to 1.44), which was highest in the first 3 months (adjusted HR = 1.75, 95% CI = 1.51 to 2.01) but also persisted 10 years or more after PC diagnosis (adjusted HR = 1.39, 95% CI = 1.28 to 1.51). Figure 1 shows adjusted HRs for anxiety disorders associated with high-risk or low- to intermediate-risk PC by time since the index date.

**Table 2.** Associations between PC diagnosis (1998-2017) and risk of anxiety disorders through 2018, Sweden

	Anxiety disorders, n		**Adjusted HR (95% CI)** ^a^	*P*
	PC cases	Controls		
**High-risk PC**				
Entire follow-up period	4280	29 056	1.96 (1.87 to 2.05)	<.001
<3 months	407	6925	2.99 (2.49 to 3.59)	<.001
3 to <12 months	658	3718	2.02 (1.78 to 2.28)	<.001
1 to <2 years	607	3575	2.27 (2.02 to 2.56)	<.001
2 to <5 years	1063	6781	1.92 (1.76 to 2.09)	<.001
5 to <10 years	1030	5665	1.88 (1.73 to 2.05)	<.001
≥10 years	515	2392	1.53 (1.35 to 1.74)	<.001
**High-risk PC (2005-2017)** ^b^				
ADT only	1617	12 127	2.08 (1.93 to 2.25)	<.001
Radiation	812	6118	1.74 (1.58 to 1.92)	<.001
Radical prostatectomy	146	1072	1.44 (1.13 to 1.84)	.003
Radical prostatectomy and radiation	85	623	1.57 (1.16 to 2.13)	.003
**Low- or intermediate-risk PC**				
Entire follow-up period	7192	53 859	1.39 (1.34 to 1.44)	<.001
<3 months	604	8996	1.75 (1.51 to 2.01)	<.001
3 to <12 months	995	6344	1.54 (1.40 to 1.70)	<.001
1 to <2 years	824	6028	1.40 (1.28 to 1.54)	<.001
2 to <5 years	1713	13 091	1.29 (1.21 to 1.38)	<.001
5 to <10 years	1858	13 058	1.35 (1.27 to 1.44)	<.001
≥10 years	1198	6342	1.39 (1.28 to 1.51)	<.001
**Low- or intermediate-risk PC (2005-2017)** ^b^				
Surveillance (no active treatment)	2936	25 859	1.29 (1.23 to 1.36)	<.001
ADT only	768	5033	1.76 (1.58 to 1.97)	<.001
Radiation	874	6392	1.44 (1.31 to 1.59)	<.001
Radical prostatectomy	456	3996	1.13 (1.00 to 1.28)	.05
Radical prostatectomy and radiation	110	1008	1.16 (0.91 to 1.49)	.24

a Adjusted for age, birth country, marital status, education, income, region, and prior history of psychiatric disorders (major depression, anxiety disorders, bipolar disorder, schizophrenia) at index date. ADT = androgen deprivation therapy; CI = confidence interval; HR = hazard ratio; PC = prostate cancer.

b Subanalysis is based on treatment data available during 2005-2017, compared with matched controls.

In men with high-risk PC, the risk of anxiety disorders varied significantly by PC treatment (*P *=* *.002) (Table 2). Those treated only with ADT (adjusted HR = 2.08, 95% CI = 1.93 to 2.25) had higher risks than those treated with radiation (adjusted HR = 1.74, 95% CI = 1.58 to 1.92), radical prostatectomy (adjusted HR = 1.44, 95% CI = 1.13 to 1.84), or both radical prostatectomy and radiation (adjusted HR = 1.57, 95% CI = 1.16 to 2.13) (*P *=* *.005, *P *=* *.005, and *P *=* *.08, respectively, for comparisons with ADT) (Table 2). Risk of anxiety disorders was significantly elevated among men treated with GnRH analogues (adjusted HR = 2.20, 95% CI = 2.02 to 2.40) or anti-androgen monotherapy (adjusted HR = 1.63, 95% CI = 1.36 to 1.96) (*P *=* *.003 for difference in HRs). Very advanced/regionally metastatic PC and distant PC metastases were more common among all men treated only with ADT (17% and 16%, respectively) than among those treated with radiation (9% and 7%), radical prostatectomy (4% and 3%), or both radiation and radical prostatectomy (5% and 4%) (4,14).

Among men with low- or intermediate-risk PC, the risk of anxiety disorders also varied by PC treatment (*P *<* *.001) (Table 2). Risk was higher among men treated only with ADT (adjusted HR = 1.76, 95% CI = 1.58 to 1.97) than those with surveillance (ie, no active treatment) (adjusted HR = 1.29, 95% CI = 1.23 to 1.36) or treated with radiation (adjusted HR = 1.44, 95% CI = 1.31 to 1.59) or radical prostatectomy (adjusted HR = 1.13, 95% CI = 1.00 to 1.28) (*P *<* *.001, *P *=* *.007, and *P *<* *.001, respectively, for comparisons with ADT).

In men either with or without a prior registration of anxiety disorder before the index date, both high-risk and low- or intermediate-risk PC were associated with significantly increased subsequent risk of anxiety disorders. In a secondary analysis, risk of new-onset anxiety disorders was assessed by excluding men with a prior registration of anxiety disorders (7184 [4%] PC cases and 81 331 [5%] controls), instead of adjusting for prior anxiety disorders in the entire cohort as in the main analyses. Most risk estimates were moderately reduced, but a nearly 3-fold risk of anxiety disorders remained in the first 3 months after diagnosis with high-risk PC (adjusted HR = 2.99, 95% CI = 2.42 to 3.70) (Supplementary Table 2, available online).

### Other secondary analyses

**Figure 2.**
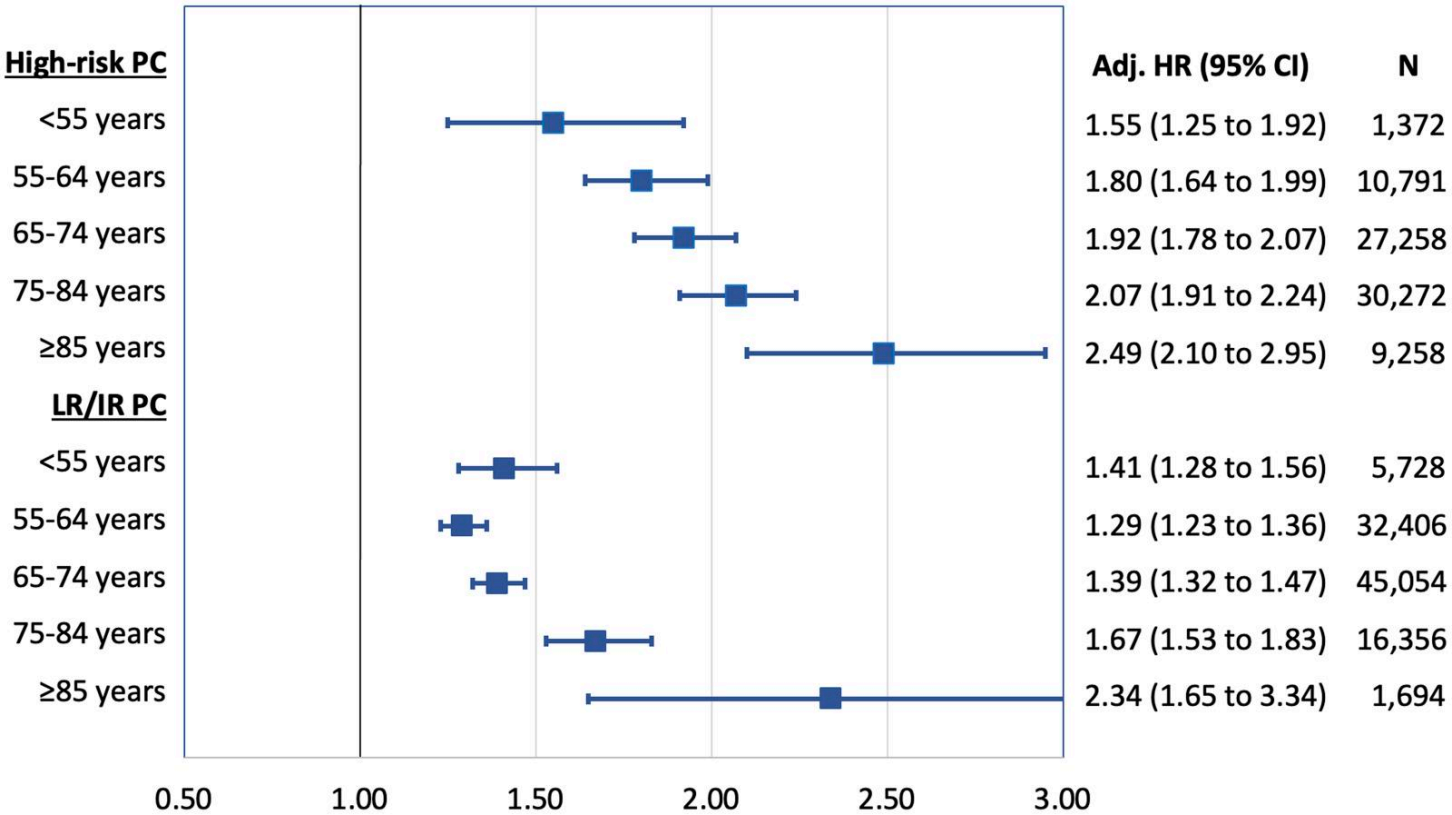
Adjusted hazard ratios (HRs) for anxiety disorders associated with high-risk prostate cancer (PC) or low- to intermediate-risk PC by age at index date, 1998-2018, Sweden. CI = confidence interval; LR/IR = low risk/intermediate risk.

Men with PC had an elevated risk of anxiety disorders regardless of age at PC diagnosis, but with significant heterogeneity by age (*P *<* *.001). For either high-risk or low- or intermediate-risk PC, the relative rate for anxiety disorders was highest in men diagnosed with PC at 85 years of age and older. Figure 2 shows adjusted HRs for anxiety disorders associated with high-risk or low-or intermediate-risk PC by age at the index date.

Stratifying on year of PC diagnosis, risk of anxiety disorders was highest in men diagnosed with high-risk PC in earlier years (1998-2004: adjusted HR = 2.92, 95% CI = 2.39 to 3.58) but remained >2-fold elevated even in later years (eg, 2010-2017: adjusted HR = 2.13, 95% CI = 1.93 to 2.35) (Supplementary Table 3, available online).

Among men with PC or controls who were diagnosed with anxiety disorders, 70% had a diagnosis registered only in primary care, and 14% had a diagnosis only in specialty outpatient or inpatient care. After restricting the outcome to anxiety disorders ever diagnosed in specialty care (30% of all cases), an increased risk of anxiety disorders persisted among men with high-risk PC (adjusted HR = 1.61, 95% CI = 1.50 to 1.73) or low- or intermediate-risk PC (adjusted HR = 1.33, 95% CI = 1.25 to 1.41).

## Discussion

In this large population-based cohort, men diagnosed with high-risk PC had nearly a 2-fold subsequent increased risk of anxiety disorders compared with control men without PC, after adjusting for sociodemographic factors and prior psychiatric diagnoses. This risk was highest in the first 3 months (nearly 3-fold) but remained significantly elevated (1.5-fold) ≥10 years after PC diagnosis. Men with high-risk PC treated only with ADT had the highest risk of anxiety disorders. In contrast, men with low- or intermediate-risk PC had a modestly increased risk of anxiety disorders.

We previously reported that men with PC had higher risks of depression (4), suicide (4), and alcohol and drug use disorders (14) compared with control men without PC. To our knowledge, the present study is the first to examine long-term risk of anxiety disorders associated with PC in a large population-based cohort. Anxiety disorders are the most common mental disorders in general populations (11,12) and have unique symptoms that may cause significant suffering and reduce function and quality of life (8-10). A previous meta-analysis with a total of 24 526 men with PC (mean sample size <500) reported that the pooled prevalence of “significant anxiety symptoms” was 17% (13). This was overall consistent with a prior meta-analysis with 4494 men with PC (mean sample size <200) that reported pretreatment, on-treatment, and post-treatment anxiety prevalences of 27%, 15%, and 18%, respectively (29). However, those studies lacked clinical diagnoses of anxiety disorders and a comparison group of men without PC, and thus were unable to estimate the risk of anxiety disorders in men with vs without PC.

The present study extends prior evidence by examining long-term risk of anxiety disorders and periods of heightened risk in different PC risk groups and by PC treatment. Men with high-risk PC had the highest risk of anxiety disorders, especially within 3 months of PC diagnosis, consistent with limited prior evidence for advanced PC stage in a small sample (30). Moreover, those treated only with ADT had the highest risk (2-fold), consistent with elevated risks of depression (4,31), anxiety (32), or suicide (4) previously reported among men treated with ADT. These findings may potentially be related to side effects of ADT (33) as well as more aggressive cancers with worse prognosis compared with men treated with radiation or radical prostatectomy. In this cohort, men treated only with ADT were more likely to have very advanced or metastatic disease than those treated with radiation or radical prostatectomy, as previously reported (4,14). We also found that the risk of anxiety disorders was elevated regardless of age at PC diagnosis, but it was highest in older men (especially ≥85 years), consistent with some (30) but not all (34,35) prior evidence.

These findings have important clinical implications. Anxiety has been associated with worse quality of life (10,36) and health outcomes (30,37) in men with PC. Follow-up care for men with PC should include close monitoring for psychosocial distress and anxiety symptoms, particularly shortly after PC diagnosis. Several instruments to screen for anxiety have been used effectively in men with PC, including the Memorial Anxiety Scale for Prostate Cancer (MAX-PC) (38), Hospital Anxiety and Depression Scale (HADS) (39), State Trait Anxiety Inventory (STAI) (40), and PROMIS Anxiety scores (41). Early detection of significant anxiety or psychosocial distress is essential for timely referral and treatment, which has been shown to improve quality of life in cancer survivors (42).

### Strengths and limitations

A key strength of this study was its large national cohort design, which provided high statistical power needed to examine PC risk groups, narrowly defined periods of susceptibility, and potential differences by PC treatment or age. The availability of diagnoses from primary care clinics where most anxiety disorders are diagnosed (27) enabled more complete ascertainment and more valid risk estimates based on a national population. We also were able to control for multiple potential confounders. Previously reported incidences of anxiety disorders and other common mental health outcomes are comparable between Sweden and the United States (11,27).

This study also had certain limitations. First, anxiety disorders were identified from ICD codes, but more detailed clinical information needed to validate them was unavailable. However, their validity has previously been supported by their prevalence, sex ratio, sibling correlations, and associations with well-documented risk factors (27). The cumulative incidence of anxiety disorders in this cohort after a median follow-up of 5 years (5%) was intermediate to the estimated 12-month (4%) and lifetime (10%) prevalence of anxiety disorders among men worldwide (12). As in other large population-based studies, diagnoses in the present cohort may represent more severe cases because mild anxiety is commonly underreported. It is possible that anxiety disorders were more likely to be identified in men with PC because of greater contact with the health-care system (ie, detection bias), particularly in the first year after PC diagnosis. Last, this study will need replication in other countries when feasible, including diverse populations, to explore for potential racial/ethnic differences.

In this large population-based cohort, men with PC had substantially increased risk of anxiety disorders, especially those with high-risk PC, ADT use, or older age. This risk was highest in the first 3 months but remained significantly elevated 10 years or more after PC diagnosis. These findings add to prior evidence showing the importance of addressing mental health in men with PC. PC survivors need close monitoring for timely detection and treatment of psychosocial distress and anxiety, particularly shortly after PC diagnosis.

## Supplementary Material

pkae087_Supplementary_Data

## Data Availability

Due to legal and ethical concerns, supporting data cannot be made openly available. Further information about the data registries is available from the Swedish National Board of Health and Welfare: https://www.socialstyrelsen.se/en/statistics-and-data/registers/.
