# Supplementary material for: Risk of anxiety disorders in men with prostate cancer: a national cohort study
Source: JNCI Cancer Spectr. 2024 Sep 14;8(5):pkae087. doi: 10.1093/jncics/pkae087 (PMC11512143; doi:10.1093/jncics/pkae087)
Supplement: pkae087_Supplementary_Data [file pkae087_supplementary_data.docx]

**SUPPLEMENTARY MATERIAL**

**Contents**

**Supplementary Table 1.** Associations between high-risk PC, stratified by locally advanced, very advanced, or distant metastases (1998-2017), and risk of anxiety disorders through 2018.

**Supplementary Table 2.** Associations between PC diagnosis (1998-2017) and risk of anxiety disorders through 2018, after excluding men with any diagnosis of anxiety disorders before index date.

**Supplementary Table 3.** Associations between high-risk PC, stratified by year of diagnosis, and risk of anxiety disorders within the next 2 years.

**Supplementary Table 1.** Associations between high-risk PC, stratified by locally advanced, very advanced, or distant metastases (1998-2017), and risk of anxiety disorders through 2018.

| **Time after PC diagnosis** | **Anxiety disorders, n** | | **Adjusted HR (95% CI)^a^** | ***P*** |
| --- | --- | --- | --- | --- |
|  | **PC cases** | **Controls** |  |  |
| **High-risk PC/locally advanced** |  |  |  |  |
| Entire follow-up period | 2,977 | 20,302 | 1.81 (1.72, 1.91) | <0.001 |
| <3 months | 245 | 4,352 | 2.49 (1.97, 3.15) | <0.001 |
| 3 to <12 months | 432 | 2,395 | 2.01 (1.73, 2.34) | <0.001 |
| 1 to <2 years | 386 | 2,372 | 1.95 (1.68, 2.26) | <0.001 |
| 2 to <5 years | 730 | 4,819 | 1.79 (1.62, 1.99) | <0.001 |
| 5 to <10 years | 770 | 4,376 | 1.76 (1.60, 1.95) | <0.001 |
| ≥10 years | 414 | 1,988 | 1.50 (1.30, 1.72) | <0.001 |
| **High-risk PC/very advanced** |  |  |  |  |
| Entire follow-up period | 794 | 5,116 | 2.45 (2.20, 2.72) | <0.001 |
| <3 months | 85 | 1,409 | 5.26 (3.53, 7.83) | <0.001 |
| 3 to <12 months | 143 | 728 | 2.30 (1.74, 3.04) | <0.001 |
| 1 to <2 years | 107 | 654 | 2.64 (2.01, 3.47) | <0.001 |
| 2 to <5 years | 216 | 1,188 | 2.40 (1.98, 2.91) | <0.001 |
| 5 to <10 years | 165 | 856 | 2.20 (1.77, 2.74) | <0.001 |
| ≥10 years | 78 | 281 | 1.87 (1.33, 2.63) | <0.001 |
| **High-risk PC/distant metastases** |  |  |  |  |
| Entire follow-up period | 509 | 3,638 | 2.31 (2.03, 2.62) | <0.001 |
| <3 months | 77 | 1,164 | 3.17 (2.06, 4.88) | <0.001 |
| 3 to <12 months | 83 | 595 | 1.69 (1.19, 2.40) | 0.003 |
| 1 to <2 years | 114 | 549 | 3.34 (2.54, 4.40) | <0.001 |
| 2 to <5 years | 117 | 774 | 1.97 (1.54, 2.54) | <0.001 |
| 5 to <10 years | 95 | 433 | 2.50 (1.88, 3.32) | <0.001 |
| ≥10 years | 23 | 123 | 1.37 (0.78, 2.42) | 0.27 |

^a^Adjusted for age, birth country, marital status, education, income, region, and prior history of psychiatric disorders (major depression, anxiety disorders, bipolar disorder, schizophrenia) at index date.

CI = confidence interval, HR = hazard ratio, PC = prostate cancer

**Supplementary Table 2.** Associations between PC diagnosis (1998-2017) and risk of anxiety disorders through 2018, after excluding men with any diagnosis of anxiety disorders before index date.

| **Time after PC diagnosis** | **Anxiety disorders, n** | | **Adjusted HR (95% CI)^a^** | ***P*** |
| --- | --- | --- | --- | --- |
|  | **PC cases** | **Controls** |  |  |
| **High-risk PC** |  |  |  |  |
| Entire follow-up period | 3,407 | 18,484 | 1.66 (1.58, 1.74) | <0.001 |
| <3 months | 195 | 729 | 2.99 (2.42, 3.70) | <0.001 |
| 3 to <12 months | 403 | 2,145 | 1.94 (1.69, 2.22) | <0.001 |
| 1 to <2 years | 455 | 2,596 | 1.97 (1.74, 2.23) | <0.001 |
| 2 to <5 years | 900 | 5,627 | 1.59 (1.46, 1.74) | <0.001 |
| 5 to <10 years | 951 | 5,121 | 1.56 (1.43, 1.70) | <0.001 |
| ≥10 years | 503 | 2,266 | 1.29 (1.14, 1.46) | <0.001 |
| **Low- or intermediate-risk PC** |  |  |  |  |
| Entire follow-up period | 5,543 | 35,738 | 1.16 (1.12, 1.20) | <0.001 |
| <3 months | 245 | 1,017 | 2.02 (1.70, 2.40) | <0.001 |
| 3 to <12 months | 519 | 3,035 | 1.39 (1.24, 1.56) | <0.001 |
| 1 to <2 years | 577 | 3,933 | 1.20 (1.08, 1.34) | <0.001 |
| 2 to <5 years | 1,366 | 10,222 | 1.07 (1.00, 1.14) | 0.05 |
| 5 to <10 years | 1,687 | 11,553 | 1.09 (1.02, 1.16) | 0.007 |
| ≥10 years | 1,149 | 5,978 | 1.13 (1.04, 1.23) | 0.003 |

^a^Adjusted for age, birth country, marital status, education, income, region, and prior history of psychiatric disorders (major depression, bipolar disorder, schizophrenia) at index date.

CI = confidence interval, HR = hazard ratio, PC = prostate cancer

**Supplementary Table 3.** Associations between high-risk PC, stratified by year of diagnosis, and risk of anxiety disorders within the next 2 years.

| **Year of PC diagnosis** | **Anxiety disorders, n** | | **Adjusted HR (95% CI)^a^** | ***P*** |
| --- | --- | --- | --- | --- |
|  | **PC cases** | **Controls** |  |  |
| 1998-2004 | 205 | 1,087 | 2.92 (2.39, 3.58) | <0.001 |
| 2005-2009 | 314 | 2,401 | 2.45 (2.06, 2.91) | <0.001 |
| 2010-2017 | 1,153 | 10,730 | 2.13 (1.93, 2.35) | <0.001 |

^a^Adjusted for age, birth country, marital status, education, income, region, and prior history of psychiatric disorders (major depression, anxiety disorders, bipolar disorder, schizophrenia) at index date.

CI = confidence interval, HR = hazard ratio, PC = prostate cancer
